# Physical Therapy Surveillance in Children with Acute Lymphoblastic Leukemia: A Quality Improvement Initiative

**DOI:** 10.3390/pediatric18020036

**Published:** 2026-03-03

**Authors:** Paula A. Ospina, Sara Fisher, Beverly A. Wilson, Lesley Pritchard, David D. Eisenstat, Cindy Fuengeling, Margaret L. McNeely

**Affiliations:** 1 Department of Physical Therapy, Faculty of Rehabilitation Medicine, College of Health Sciences, University of Alberta, Edmonton, AB T6G 2G4, Canada; pospina@ualberta.ca (P.A.O.); lwiart@ualberta.ca (L.P.); cindy.fuengeling99@gmail.com (C.F.); 2 Pediatric Oncology Unit, Stollery Children’s Hospital, Edmonton, AB T6G 2B7, Canada; sara.fisher@albertahealthservices.ca (S.F.);; 3 Department of Pediatrics, Faculty of Medicine and Dentistry, University of Alberta, Edmonton, AB T6G 2R3, Canada; eisensta@ualberta.ca; 4 Children’s Cancer Centre, Royal Children’s Hospital, Parkville, VIC 3052, Australia; 5 Department of Paediatrics, University of Melbourne, Parkville, VIC 3052, Australia; 6 Department of Oncology, Faculty of Medicine and Dentistry, University of Alberta, Edmonton, AB T6G 2R3, Canada; 7 Cancer Care Alberta, Alberta Health Services, Edmonton, AB T5J 3E4, Canada

**Keywords:** physical therapy, children, cancer, surveillance, assessment

## Abstract

Background/Objectives: Children with acute lymphoblastic leukemia (ALL) often experience treatment-related side effects. Physical therapy (PT) surveillance programs are helpful in identifying impairments; however, they do not typically incorporate assessments for peripheral neuropathy, motor proficiency, and foot drop. Our aim is to explore the feasibility of conducting additional functional tests to an existing surveillance program to improve the identification of impairments and characterize the prevalence of treatment-related deficits in children with ALL. Methods: A prospective, longitudinal descriptive study, embedded into a quality improvement initiative, was conducted. The surveillance program included standard assessments for ankle range of motion, activity level, balance, functional capacity, pain, gait, and kneeling to standing. Additional tests included motor and sensory function, foot posture, motor performance, quality of life, feasibility (recruitment and completion rates), service provision, and self-reported symptoms. Data were collected over 3 months. Results: Twenty children completed the study and 19 completed all assessments. Nineteen children presented deficits in at least two physical function tests. The most prevalent deficit identified from standard PT tests included decreased ankle range of motion (*n* = 19; 95%), and the most common deficit seen in the additional tests was impaired motor and sensory function (*n* = 14/19; 74%). Pain was the most common self-reported symptom in the checklist and the second worst subscale score in the pain dimension of the quality of life questionnaire (*p* < 0.001). Conclusions: Several treatment-related deficits were identified in children with ALL. Further research is warranted to explore the use of a standardized symptom checklist for the timely identification of functional limitations and impairments.

## 1. Introduction

Acute lymphoblastic leukemia (ALL) is the most common childhood cancer, representing approximately 25% of cancers in children aged up to 15 years [1]. In Canada, it is estimated that 205 children under 15 years of age will be diagnosed with ALL each year [2]. Advancements in cancer treatments and early diagnosis have improved ALL outcomes, with a 5-year survival rate of 94% [2,3]. Increased survival rates have resulted in a larger number of children living with treatment-related side effects such as decreased range of motion [4,5,6,7], muscle weakness [4,5], balance deficits [8,9], decreased gross and fine motor performance [4,10,11,12], gait deficits [13], and chemotherapy-induced peripheral neuropathy (CIPN) [14,15].

CIPN, a common side effect in children receiving neurotoxic agents as part of the ALL treatment, can cause pain, numbness, and weakness, manifesting first in the lower extremities in a stocking pattern, followed by the upper extremities in a glove pattern of distribution [16,17,18,19]. Research shows high prevalence rates of CIPN in children and adolescents during the first year of ALL treatment, with a prevalence peak in the first 2 to 4 months [14], and deficits lasting years after treatment [5,20,21,22]. CIPN is known as one of the most distressing symptoms experienced by patients [17], as the sequelae resulting from this condition can lead to limitations in daily activities and restrictions in participation, negatively affecting the quality of life of children and adolescents with cancer [23].

Although functional mobility often improves by the completion of cancer treatment, childhood cancer survivors continue to demonstrate lower functional mobility than age-matched norms, with persistent deficits seen in 5% to 54% of survivors [4,11,24,25]. Children with ALL can present with decreased passive and active range of motion (ROM) in ankles and hips [4,5,26], and impaired walking capacity [5,7] years after chemotherapy completion. Sequelae from ALL treatment regimens may lead to long-term deficits that impact physical function [5], and contribute to frailty in adulthood, which is associated with the onset of chronic health conditions at a younger age [27,28]. Traditional models of care that limit rehabilitation until after patients complete cancer treatment or when severe functional declines occur result in long-term physical and psychological sequelae in cancer survivors [29]. Therefore, children with ALL require close monitoring and timely intervention approaches for cancer therapy side effects as these can persist or develop months or years after the completion of cancer treatments [30].

The Italian Association of Pediatric Hematology and Oncology published a consensus of recommendations on the role of rehabilitation in children with cancer, advocating for physical rehabilitation from the time of diagnosis through all phases of treatment and recovery to reduce treatment-related toxicities, support functional independence, and improve quality of life [30,31,32]. Therefore, childhood cancer survivors should be assessed from diagnosis to identify the (1) cancer treatment plan, (2) clinical and psychosocial conditions of the patient, (3) family context, and (4) rehabilitative needs to define the rehabilitation program [30]. The consensus also recommends that children be screened regularly for rehabilitative needs, especially when there is a high risk of treatment-related toxicity, to assess the results of the rehabilitation plan, track persisting problems, and update the goals of the program based on the patient’s clinical condition, needs, and stage of cancer treatment [29,30,32].

The Stollery Children’s Hospital in Edmonton, Canada, introduced a surveillance physical therapy (PT) program in 2020 to monitor the impact of ALL treatment on the physical function of children. This program aims to monitor physical deficits during ALL treatment and support the timely referral to rehabilitation services for children presenting with physical and functional deficits. To date, the program has enabled the early identification of physical deficits such as muscle weakness, decreased ankle mobility, impaired balance, decreased functional capacity, impaired gait, and the timely referral to rehabilitation services. However, the surveillance battery does not include assessments to detect CIPN, or to monitor for motor development and foot posture, domains commonly affected by ALL chemotherapy treatment.

We proposed a quality improvement initiative to (1) explore the feasibility of conducting tests to assess CIPN, motor proficiency, and foot drop in children with ALL as part of the surveillance PT program, and (2) describe the prevalence of treatment-related deficits experienced by children with ALL at the Stollery Children’s Hospital.

## 2. Materials and Methods

### 2.1. Study Design

A prospective, longitudinal descriptive study was embedded into a quality improvement initiative at the hospital to inform the design and feasibility of a future interventional study. Children aged 4 to 17 years, diagnosed with ALL, were recruited at the Stollery Children’s Hospital. Data from PT assessments were collected over a 3-month period.

### 2.2. Sampling and Participants

A convenience sample of children (1) diagnosed with ALL aged between 4 and 17 years (at time of diagnosis) and their parent or primary caregiver; (2) scheduled to start the consolidation phase or undergoing the maintenance phase of the ALL treatment protocol established by the Children’s Oncology Group; (3) having no history of a neurodevelopmental or genetic disorder (i.e., Down syndrome, autism, seizure disorder) prior to the cancer diagnosis; and (4) able to speak and comprehend English were eligible to participate in the study.

A consecutive sampling approach was used until a minimum sample size of 20 eligible children was reached. The target sample size of 20 was estimated based on the August 2020 summary report of the surveillance program, including the number of children starting the program who were newly diagnosed with ALL (*n* = 13) and those undergoing the maintenance phase of ALL therapy (*n* = 14). Given known challenges in recruitment of children with cancer, a sample size of 20 was deemed appropriate to evaluate study feasibility.

### 2.3. Procedures

Potential participants were identified at the children’s oncology clinic following referral from the oncology team. Parents and caregivers were provided with study information and, upon expressed interest, were contacted by the investigators to confirm study eligibility, review study details, and respond to questions. The parents/legal guardians of eligible participants provided written consent for their child to take part in the study.

Following enrollment in the study, parents and caregivers of participating children received a link to a secure Research Electronic Data Capture (REDCap) service, a secure electronic data capture tool hosted and supported by the Women and Children’s Health Research Institute at the University of Alberta [33]. Participants completed questionnaires capturing demographic and medical information, self-reported symptoms, current PT service provision, and health-related quality of life (HRQOL). Over a period of three months, children underwent PT assessments at scheduled clinic visits and parents provided consent for the study team to access and use the evaluation data for the study.

### 2.4. Data Sources

Data on physical function assessments measured by the designated pediatric oncology physical therapist in the oncology department were collected including: *ankle ROM*, using a goniometer [34]; *activity level*, using the Lansky Play-Performance Scale [35]; *balance*, using the single-leg stance test [36]; *functional capacity*, using the Six-minute Walk Test (6MWT) [37]; *pain*, using the Wong–Baker Faces Pain Rating Scale [38]; *gait*, using the observational gait analysis [39]; *kneeling to standing*, using the half-kneel to stand from the floor test [40];) *motor and sensory function*, using the pediatric-modified Total Neuropathy Scale (ped-mTNS) [41]; *foot posture*, using the Foot Posture Index (FP1-6) [42]; and *motor performance*, using the Bruininks–Oseretsky Test of Motor Proficiency version II (BOT-2)—short form [43] (Document S1).

Additional outcomes to standard clinical metrics included *HRQOL*, using the Pediatric Quality of Life Inventory (PedsQL) Parent Proxy-Cancer Module [44]; *feasibility*, with recruitment rates and completion rates of measurements; and *service provision*, with referral and attendance rates. Data from questionnaires about the child’s symptoms, impairments, current PT service provision, and HRQOL were collected at baseline, 1 month, 2 months, and 3 months. Throughout the study, we collected data on completion and recruitment rates.

### 2.5. Statistical Analysis

Descriptive statistics by categorical factors were calculated for demographic data, medical treatment characteristics, HRQOL, motor and sensory function, motor performance, and foot posture. Data are presented as mean ± standard deviation (SD), median, range, and/or percentage according to the variable type. Box plots were created where appropriate.

#### 2.5.1. Recruitment Rates

Recruitment rates were calculated by dividing the number of children/families enrolled in the study by the number of children/families approached to participate in the study. Reasons for not participating in the study were categorized and presented as frequencies.

#### 2.5.2. Completion Rates

Completion rates were calculated by dividing the number of fully completed assessments, by the number of planned assessments. Reasons for not completing the assessments were categorized and presented as relative frequencies (percentages).

#### 2.5.3. Physical Function Outcomes

Physical function outcome data are presented as relative frequencies (percentages) for each variable. For motor performance, the BOT-2 standard scores were converted to *z*-scores corresponding to the performance of age-matched norms of typically developing children and were presented in a box plot.

#### 2.5.4. HRQOL Outcomes

The Kruskal–Wallis non-parametric test was used to evaluate whether the distribution of scores over time differed for each subscale. We also performed a one-way ANOVA followed by the post hoc Dunnett’s test to estimate the significance of the difference between the mean of each PedsQL subscale and the mean of the subscale with the lowest PedsQL score. The Bonferroni correction was used to adjust the significance level as we performed 6 *post hoc* comparisons (=0.05/6 = 0.007). We reported the estimated *p*-value and highlighted in bold the subscales with a *p*-value < 0.007.

#### 2.5.5. Self-Reported Symptoms and Deficits

Symptoms and deficits were categorized and presented as relative frequencies (percentages).

#### 2.5.6. Service Provision

Data are presented as percentages for children (1) referred to rehabilitation services who participated in the sessions; (2) referred to rehabilitation services who did not participate in the sessions; and (3) number of sessions attended. Reasons for not attending rehabilitation services were categorized and presented as percentages.

## 3. Results

A total of 32 children were screened for eligibility. Of the 28 eligible children, 21 parents/guardians consented to participate. After consenting, one participant did not complete the questionnaires, and the physical therapist was unable to complete any assessments due to health issues with the child. Therefore, a total of 20 children completed the study and were included in the analysis.

Demographic and medical characteristics are presented in Table 1. The mean age of children at the time of enrollment in the study was 8.24 years (range 4–16 years). The majority of children were males (*n* = 14; 70%), diagnosed with B-cell ALL (*n* = 17; 85%), with a standard risk level (*n* = 12; 60%), undergoing the maintenance phase of ALL therapy (*n* = 16; 80%), and who did not present any concurrent medical conditions (*n* = 19; 95%). The location of residential living varied, with *n* = 12 (60%) families living in the Edmonton Metropolitan Area and *n* = 8 (40%) from other locations within the province.

### 3.1. Feasibility Outcomes

#### 3.1.1. Recruitment Rates

A total of 75% of eligible participants were enrolled in the study. Of the 32 children screened for eligibility, four did not meet the eligibility criteria, and seven potentially eligible children declined to take part in the study. Reasons for not participating included parent overwhelmed (*n* = 3), moving out of province (*n* = 1), previous bad experience with research (*n* = 1), prolonged isolation at hospital (*n* = 1), and child not compliant with regular PT assessments (*n* = 1).

#### 3.1.2. Completion Rates

All study participants completed 100% of the standard PT surveillance tests, and 90% (*n* = 18) completed all the additional PT surveillance tests, resulting in a completion rate of 97%, exceeding our target feasibility rate of ≥80%. We were unable to administer the ped-mTNS test to one participant due a potential diagnosis of selective mutism, limiting the child’s ability to communicate with the therapist during the test. We were also unable to complete the vibration subtest from the ped-mTNS test with another participant, as the child could not sense vibration at any intensity, and it was unclear if this reflected a true sensory deficit or issues with the child’s understanding of the assessment.

### 3.2. Physical Function Outcomes

A total of 19 (95%) children had moderate and/or severe deficits in at least two physical function tests and only *n* = 1 out of 20 participants showed no physical function deficits within the 3-month period. Of the 10 tests, the average number of physical function outcomes showing deficits was 5.3 ± 2.49, with a range from 0 to 9 tests indicating the high presence of deficits at a given assessment.

Results will be presented in two separate sections: the first section reports the prevalence of deficits identified using standard physical function outcomes, and the second section reports deficits identified using additional physical function tests incorporated in the PT surveillance program.

#### 3.2.1. Prevalence of Deficits in PT Surveillance Standard Physical Function Outcomes

The frequency of administration of the standard physical function assessments varied among participants as the selection of tests was at the discretion of the physical therapist and was determined based on the (1) reported symptoms and deficits seen in the child at the time of the evaluation; (2) frequency of hospital visits of the child; (3) physical therapist’s capacity to complete the assessments with the resources in place; (4) scheduled medical procedures and tests; (5) family availability; and (6) presence of symptoms (i.e., fatigue, headache, pallor, bruising, petechia, abnormal bleeding, fever, abnormal vital signs, nausea, or a new rash) [45].

Results from the physical function outcomes at the initial assessment, follow-up assessment, and classification status (i.e., the number of participants that improved, declined or maintained from the initial assessment to follow-up) are presented in Table 2.

The standard physical function outcomes in the PT surveillance program with the highest prevalence of deficits at the initial assessment were ankle dorsiflexion ROM with a total of *n* = 19 (95%) and *n* = 17 (85%) participants showing deficits in active and passive ROM, respectively. This was followed by balance impairments, with *n* = 14 (70%) children and adolescents presenting moderate and/or severe deficits during the single-leg stance test, and reduced functional capacity, with 12 participants (60%) exhibiting impaired functional aerobic capacity and endurance in the 6MWT (Table 2).

The outcomes with the lowest prevalence of physical function deficits at the initial assessment were activity level (Lansky Play-Performance Scale), with only *n* = 3 (15%) children presenting moderate deficits carrying out daily activities, and pain, with *n* = 4 (20%) participants reporting moderate levels of pain.

The outcomes with the most follow-up assessments were gait and ankle ROM with up to *n* = 16 (80%) and *n* = 12 (60%) children being reassessed at multiple time points, respectively. Ankle ROM was also the outcome with the highest prevalence of children presenting severe deficits at the initial assessment as well as with a worsening of scoring classification.

#### 3.2.2. Additional Physical Function Outcomes

The physical function tests added to the PT surveillance program, including the BOT-2 (short form), FP1-6, and ped-mTNS were assessed at only one point within the three-month period. Results from these tests are shown in Table 3 and Table 4.

Of the three additional physical function outcomes included in the PT surveillance program, the outcome with the highest prevalence of deficits was motor and sensory function (ped-mTNS), with *n* = 14/19 (74%) participants showing scores indicative of CIPN; followed by motor performance (BOT-2 short form), with *n* = 11 (55%) children presenting moderate and/or severe deficits in fine and gross motor skills; and foot posture (FP1-6), with *n* = 10 (50%) participants presenting foot pronation and/or overpronation in one or both feet (Table 3).

**Figure 1:**
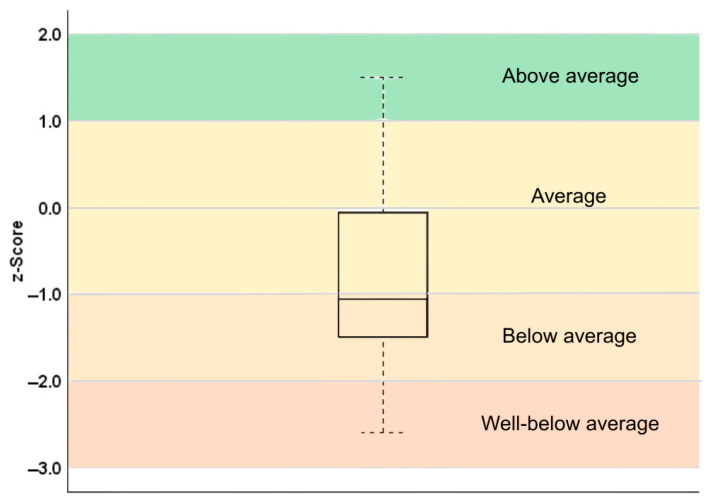
BOT-2 scores compared to the performance of age-specific norms.

Motor performance: Results from the converted BOT-2 (short form) standard scores to *z*-scores corresponding to the performance of age norms of typically developing children show that over 50% of participants were -1SD below the mean compared to typically developing age norms (Figure 1, Table 3).

Foot posture: Results from the FP1-6 test scores ranged between 0 and 10, or between neutral foot posture and foot overpronation (Table 4).

Motor and sensory function: The ped-mTNS domains with the most common deficits seen in children were light touch (1.68 ± 1.41) and deep tendon reflexes (1.47 ± 1.46), affecting 68% and 74% of the participants, respectively (Table 3 and Table 4). The least affected ped-mTNS domain was vibration (0.16 ± 0.5) with only *n* = 2/18 (11%) children presenting with deficits.

### 3.3. HRQOL Outcomes

A total of *n* = 19 (95%) children presented with at least one low PedsQL total score, and 45% (*n* = 9) had at least one total score in the severe range across the 3-month period, based on the clinically meaningful interpretation of PedsQL scores proposed by Beverung et al. (2025) [46]. Descriptive statistics for the PedsQL scores for all time points are presented in Table 5. The distribution of score values of the PedsQL total scores and subscale scores is available in Figure S1.

We tested whether the distributions of PedsQL scores for all subscales over time differ in each subscale. Overall, no statistical differences were observed over time (*p* > 0.05); the distribution of the scores was consistent over time in each category (Table S1).

After testing whether the PedsQL subscales differ in relation to the PedsQL subscale with the lowest score, results indicated that procedural anxiety, pain and hurt, and nausea were the subscales with the lowest HRQOL scores, whereas treatment anxiety, worry, and physical appearance were the subscales with the highest HRQOL scores (*p* < 0.001) (Table 6).

### 3.4. Self-Reported Symptoms and Deficits

**Figure 2:**
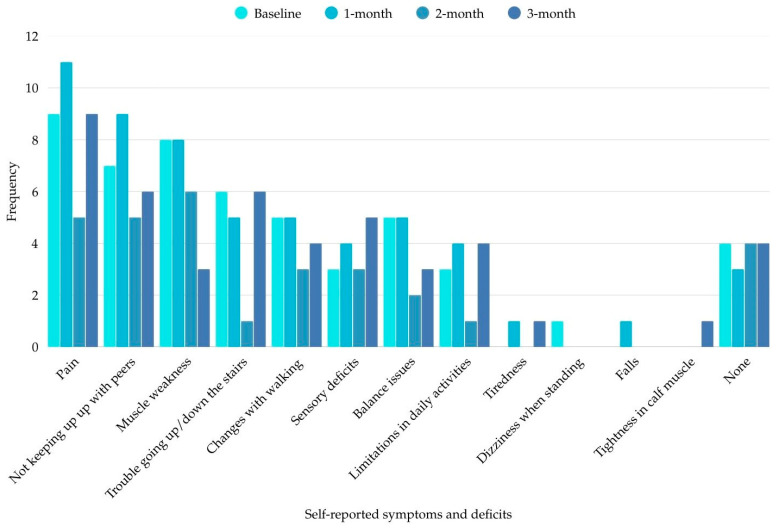
Frequency of self-reported symptoms and deficits across all time points.

A total of 19 participants (95%) reported at least one symptom or deficit resulting from the child’s ALL treatment at any of the assessment time points. The average number of symptoms and deficits reported by each participant across all time points was 2.1 ± 2.14, with a highest of *f* = 8 symptoms and deficits reported at a given time point. The most commonly reported symptoms and deficits included pain (*f* = 34), followed by not being able to keep up with friends/siblings (e.g., slower runner) (*f* = 27), and muscle weakness (*f* = 25) (Figure 2).

### 3.5. Service Provision

Of the 20 children enrolled in the study, only *n* = 6 (30%) were referred to a PT rehabilitation program. Of the six children referred to PT, five accessed the service and one was unable to attend as the referral was to a school-based program that had not yet commenced for the term. Among those who accessed PT, the total number of PT sessions attended ranged from 1 to 6 sessions.

## 4. Discussion

This quality improvement initiative evaluated the feasibility of incorporating additional physical function tests and described the prevalence of treatment-related deficits in a cohort of children diagnosed with ALL at the Stollery Children’s Hospital. Consistent with findings from a prospective PT surveillance and intervention program in children with cancer [10], the ped-mTNS, BOT-2 (short form), and FP1-6 tests were feasible outcome measures within the hospital’s PT surveillance program, with 95% of participants (*n =* 19) completing all the assessments over the 3-month evaluation period.

The BOT-2 (short form) was useful for the assessment of motor proficiency in children with ALL; however, total scores often overestimated overall proficiency because a higher performance in some subdomains masked clinically meaningful deficits in other areas. Also, the results offered limited detail regarding the child’s motor deficits, limiting the test’s usefulness for prescribing PT interventions. Although evidence suggests that the BOT-2 (short form) systematically underestimates motor proficiency levels compared to the BOT-2 (complete form) [47], our findings are consistent with prior research suggesting that the BOT-2 (short form) is primarily a screening tool with less sensitivity for detecting mild impairments [48]. In addition, composite scores do not distinguish between fine and gross motor scores, which is a limitation when prescribing PT treatment plans [49,50]. Our rationale for testing the feasibility of the BOT-2 (short form) over the BOT-2 (complete form) was due to the existing barriers in our hospital setting such as the lack of a dedicated space and time to conduct the PT assessments in the oncology department. For clinical settings where such barriers are also present, it is suggested to choose specific subtests of the BOT-2 (complete form) that will provide more practical information on specific presenting impairments, helping the physical therapist tailor the PT interventions to the identified deficits.

Decreased ankle ROM was the most prevalent deficit seen in the study, with up to 95% of children showing deficits in active dorsiflexion ROM. This finding aligns with previous studies showing a high prevalence of ankle ROM deficits in this population [4,5,6,7]. Decreased ankle ROM, which often leads to deficits in gait efficiency and energy expenditure [13], is associated with the administration of neurotoxic chemotherapeutic agents (e.g., vincristine and intrathecal methotrexate) [5], and is considered a CIPN motor impairment [17]. This is consistent with results from our study showing motor and sensory function as a prevalent deficit, with 74% (*n* = 14/19) of children presenting with CIPN. This finding highlights the importance of assessing the presence of CIPN in children with ALL, especially if ankle ROM deficits are present [51]. Research supports that all children with ALL receiving vincristine should undergo a formal assessment for CIPN [18,51,52], as an early identification of CIPN deficits can prevent a further worsening of the impairments (e.g., foot drop), minimize the adverse effects on physical function and activity participation, and can inform physical therapists on appropriate treatment plans [51]. Additionally, conducting objective CIPN assessments in children exposed to neurotoxic chemotherapy can enhance our understanding of the natural progression of this condition [51], inform oncology providers about the presence of neurotoxicity, and, in severe cases, support medical decisions such as dose reduction or delay [53].

A particular finding of this study was the discrepancy between the frequency of pain reported at the PT assessments compared to the frequency of pain reported in the self-report checklist of symptoms and deficits and the PedsQL questionnaire. While only 20% of children reported moderate levels of pain using the Wong–Baker Faces Pain Rating Scale at their initial PT assessment, pain was the most common self-reported symptom in the checklist and the second worst subscale score in the PedsQL parent-proxy questionnaire (*p* < 0.001). The latter findings align with research demonstrating the high prevalence of pain in children with ALL [14,28,54,55,56]. Approximately 50% of children with ALL experience treatment-related pain [57], and twice as many experience chronic pain (33%) compared to the general population (15–25%) [14,28,54]. Children with ALL are more vulnerable to chronic pain due to the administration of treatment-related medications such as vincristine negatively impacting the nervous system (e.g., CIPN), and dexamethasone the musculoskeletal system (e.g., myopathy) [58]. Although pain is less prevalent and distressing as treatment progresses to the less intense phases of ALL therapy, pain remains higher when compared to pre-treatment levels [59], and often persists long after the completion of ALL treatment [54,55,56]. We hypothesize that the discrepancy in pain reporting in this study reflects differences in the recall period and construct assessed by each measure. The Wong–Baker Faces Pain Rating Scale rates the intensity of pain at the time of assessment, and many children were not experiencing pain during the PT evaluation. In contrast, the PedsQL assesses the extent to which joint and/or muscle aches and severe pain have been a problem, while the self-report checklist captures symptoms experienced in the past four weeks. This discrepancy was likely influenced by several different factors including differences in parent versus child reporting, limitations due to single-time-point pain scales, and an under-recognition of symptoms during PT encounters. This finding demonstrates the need for a multidimensional, pain-specific clinical approach to better understand the nature of pain in this population, and to optimize evidence-based strategies for pain management [60]. Pain is a complex and multifactorial experience, influenced by biological, psychological, and socioecological factors [61].

Most children in our study (*n* = 19; 95%) presented at least one low HRQOL score in the 3-month period. This finding is consistent with research indicating that children with ALL experience a lower HRQOL compared to their healthy peers [62], with evidence demonstrating that factors such as fatigue and weakness can negatively influence the HRQOL of children [63]. Therefore, the timely identification and management of treatment-related deficits may help improve the HRQOL of ALL childhood cancer survivors. Unfortunately, only 30% of the children in this study were referred to a formal PT program. This is likely due to the limited resources in our hospital setting, where only children with severe physical deficits from cancer treatment are being referred to PT intervention. These results are consistent with previous studies showing that few children with cancer are referred to PT services [64,65], and also few access the service upon referral [64,66,67,68]. Many families of children with cancer experience several barriers to accessing healthcare services such as time constraints, financial burden, geographical distances, and accessibility limitations [69,70,71,72,73,74,75]. Therefore, it is important to not only ensure the early identification of treatment-related deficits and timely referral of children with ALL to PT services, but to understand the families’ capacity to access services [75]. Offering families different options to best accommodate their needs, such as PT treatments in conjunction with oncological appointments, referrals to physical therapists in the community [76,77], or virtual delivery, may help overcome some barriers to accessing rehabilitation services [75]. Alternatively, providing families with targeted PT resources (e.g., home-based programs) and ideas to engage children in an active lifestyle during daily routines can help minimize the burden of additional healthcare appointments [75].

We found the self-report checklist of symptoms and deficits to be a valuable screening tool that can guide physical therapists in the identification of treatment-related deficits. We noticed that parents reported more symptoms and deficits in the checklist compared to those reported during the PT assessments. We hypothesize that when parents are asked for specific symptoms and deficits, as opposed to an overall perception of the child’s health condition during consultation, it helps them to recall any changes in the child’s health condition and behavior. Additionally, the adoption of this screening tool may serve as a resource to educate parents of newly diagnosed children with ALL of the common deficits seen in this population, so they can inform the oncology team of any potential issues in the child that can be addressed promptly or help prevent a further worsening of the condition. To our knowledge, no evidence-based screening tools to identify treatment-related deficits in children with ALL have been published. Further research should be conducted to examine the use of a standardized symptom and deficit checklist for children with ALL that can help physical therapists tailor the assessments to the child’s presenting deficits, optimizing the time and resources in the clinical settings.

The findings of this study reflect experiences commonly reported by individuals treated for pediatric cancer, where chemotherapy-related side effects such as CIPN are routinely identified but not necessarily addressed through targeted rehabilitation. While PT is highly valued in pediatric oncology care, it may not focus sufficiently on the prevention or management of treatment-related neuromuscular impairments. Persistent functional limitations, such as impaired gait, that can persist for years after treatment, highlight the potential value of earlier, impairment-specific PT and patient education. The results of the study underscore the importance of proactively addressing chemotherapy-related side effects to reduce long-term disability and improve survivorship outcomes.

### 4.1. Limitations

This study presented several limitations. First, variability in the frequency of PT assessment administration across participants resulted in multiple missing time points, limiting analyses to descriptive statistics (frequencies and percentages). Second, to our knowledge, there are no scoring thresholds for classifying severe CIPN in the ped-mTNS test, limiting our ability to objectively identify children with severe neuropathy. A scoring criterion indicating the severity of CIPN may suggest the need for (1) increased monitoring of the condition, (2) further testing to investigate the main area(s) affected (e.g., muscle strength), and (3) early PT management to prevent a further worsening of the deficits. Third, the small sample size of children undergoing the consolidation phase or intensive treatment phases (versus maintenance phases) limited our ability to analyze data by sub-groups and inform the prevalence of physical function deficits by chemotherapy phase. Fourth, given that assessments were conducted opportunistically and at varying stages of treatment across the 3-month period, results may have been influenced by treatment phase-specific factors and the child’s health condition at the time of the assessment. Therefore, it is not possible to make inferences about the overall child’s physical function. Fifth, the relatively short period of time spent collecting data from the PT surveillance program resulted in some physical function outcomes being assessed only at one point in time due to (1) limitations in the hospital setting (e.g., time, physical therapist’s capacity, child’s scheduled medical procedures); (2) the hospital’s standard of care guidelines recommending a reassessment every six months or upon progression in the phases of ALL therapy; or (3) a recent assessment prior to enrolling in the study. Sixth, the quality improvement research design limits the ability to generalize results to other healthcare settings due to the tailoring of procedures specific to our local context (e.g., the timing of PT assessments embedded into standardized hospital protocols), the lack of a control group, and the small sample size.

### 4.2. Future Directions

Results from this quality improvement initiative helped to inform actionable changes in the PT surveillance program at the hospital including the adoption of the ped-mTNS, BOT-2 (relevant subtests), FP1-6, and self-report checklist of symptoms and deficits as part of the current standard of care services. Although a recent clinical practice guideline and expert consensus recommendations for rehabilitation in childhood cancer survivors support PT referral and assessment for children with symptomatic neuropathy [52], higher-quality research evidence supporting the benefits of PT in children with cancer is needed to create guidelines and recommendations to inform pediatric oncology PT interventions [64]. In addition to gaps in research, limited clinical resources [64] and family capacity constraints [75] are key factors that may influence referral to, and access of children with ALL to, rehabilitation services—areas that warrant further research.

A 3-month hybrid, in-person and online proof-of-concept PT program for children with ALL at the hospital—informed by the results of this study—is being conducted to investigate if this approach is feasible to improve access to PT services [78].

## 5. Conclusions

Several treatment-related deficits were identified in children with ALL, with a higher prevalence of deficits in ankle dorsiflexion ROM, CIPN, and balance. The BOT-2, ped-mTNS, and FP1-6 tests are feasible outcome measures to monitor the deficits in ALL childhood cancer survivors. Further research is warranted to explore the use of a standardized symptom and deficit checklist for an early identification of impairments and to guide the PT assessments.

## Figures and Tables

**Table 1:** Summary of participant characteristics.

Demographics	Overall *N* = 20, 100%
Child Age Upon Enrollment (Mean, SD, range)	
Age, years	8.24 (3.42) 4–16 years
Biological Sex (*n*, %)	
Female	6 (30%)
Male	14 (70%)
Height (Median, range)	
Centimeters, cm	135.9 (105.3–177.2 cm)
Weight (Median, range)	
Kilograms, kg	30.5 (19–95.6 kg)
Body Mass Index (BMI) (Mean, SD, range)	
BMI	19.70 (5.24) (14.69–33.79)
Primary Carer’s Current Employment Status (*n*, %)	
Not employed (disability)	2 (10%)
Homemaker	8 (40%)
Full Time	9 (45%)
Temporarily Unemployed	2 (10%)
Annual Family Income (*n*, %)	
<$CAD 60,000	4 (20%)
>$CAD 60,000	13 (65%)
Prefer not to answer	3 (15%)
Type of Residential Area (*n*, %)	
Edmonton Metropolitan Area	12 (60%)
Alberta/Northwest Territories rural and remote residence	8 (40%)
Primary Carer Living in the Same Residence as Child (*n*, %)	
Yes	20 (100%)
Type of Acute Lymphoblastic Leukemia (*n*, %)	
B-cell	17 (85%)
T-cell	3 (15%)
Children’s Oncology Group Risk Level (*n*, %)	
Standard	12 (60%)
High	8 (40%)
Chemotherapy Treatment Phase (*n*, %)	
Consolidation	4 (20%)
Maintenance	16 (80%)
Additional Concurrent Medical Conditions (*n*, %)	
High Blood Pressure	1 (5%)
None	19 (95%)
Past Medical Conditions (*n*, %)	
Seizures	2 (10%)
Congenital heart malformation	1 (5%)
Liver impairment	1 (5%)
Bilateral inguinal hernia	1 (5%)
None	15 (75%)
Cumulative Vincristine Dosage (*n* = 19) (Median, range)	
Dosage, mg/m^2^	28.4 (16.4–58 mg/m^2^)

**Table 2:** Summary of results of standard physical function outcomes.

Outcomes (*N* = 20)	Score Classification at Initial Assessment *N* (%)	Initial Assessment Classification of Participants with a Follow-Up Assessment *N* (%)	Change in Score Classification for Participants with a Follow-Up Assessment *N*; ↑ ↓ =
Ankle dorsiflexion ROM			
AROM (Right)	Normal 4 (20%) Moderate 12 (60%) Severe 4 (20%)	Normal 1 (5%) Moderate 8 (40%) Severe 3 (15%)	Normal 3 ↑ Moderate 2 ↓ Severe 7 ↑
AROM (Left)	Normal 4 (20%) Moderate 12 (60%) Severe 4 (20%)	Normal 2 (10%) Moderate 7 (35%) Severe 3 (15%)	Normal 4 ↑ Moderate 3 ↓ Severe 5 ↑
PROM (Right)	Normal 7 (35%) Moderate 10 (50%) Severe 3 (15%)	Normal 4 (20%) Moderate 5 (25%) Severe 3 (15%)	Normal 2 ↓ Moderate 5 = Severe 5 ↑
PROM (Left)	Normal 7 (35%) Moderate 11 (55%) Severe 2 (10%)	Normal 3 (15%) Moderate 7 (35%) Severe 2 (10%)	Normal 4 ↑ Moderate 4 ↓ Severe 4 ↑
Lansky scale	Normal 17 (85%) Moderate 3 (15%) Severe 0	Normal 5 (25%) Moderate 0 Severe 0	Normal 4 ↓ Moderate 1 ↑ Severe 0 =
Single-leg balance test			
Right	Normal 8 (40%) Affected 12 (60%)	Normal 3 (15%) Affected 6 (30%)	Normal 5 ↑ Affected 4 ↓
Left	Normal 9 (45%) Affected 11 (55%)	Normal 5 (25%) Affected 4 (20%)	Normal 6 ↑ Affected 3 ↓
FACES pain scale	Normal 16 (80%) Moderate 4 (20%) Severe 0	Normal 8 (40%) Moderate 2 (10%) Severe 0	Normal 7 ↓ Moderate 3 ↑ Severe 0 =
Observational gait analysis			
Base of support	Normal 15 (75%) Moderate 5 (25%) Severe 0	Normal 11 (55%) Moderate 5 (25%) Severe 0	Normal 10 ↓ Moderate 6 ↑ Severe 0 =
Foot progression	Normal 13 (65%) Moderate 6 (30%) Severe 1 (5%)	Normal 10 (50%) Moderate 6 (30%) Severe 0	Normal 10 = Moderate 5 ↓ Severe 1 ↑
Gait speed	Normal 15 (75%) Moderate 5 (25%) Severe 0	Normal 11 (55%) Moderate 5 (25%) Severe 0	Normal 12 ↑ Moderate 4 ↓ Severe 0 =
Proximal strength	Normal 19 (95%) Moderate 1 (5%) Severe 0	Normal 15 (75%) Moderate 1 (5%) Severe 0	Normal 14 ↓ Moderate 2 ↑ Severe 0 =
Half-kneel to stand	Normal 9 (45%) Moderate 11 (55%) Severe 0	Normal 0 Moderate 3 (15%) Severe 0	Normal 1 ↑ Moderate 2 ↓ Severe 0 =
6MWT	Normal 8 (40%) Moderate 9 (45%) Severe 3 (15%)	-	-

Score classification ranges for each test are available in Document S1. Nomenclature: ↑, increased; ↓, decreased; =, maintained. Abbreviations: AROM, Active Range of Motion; PROM, Passive Range of Motion; 6MWT, Six-minute Walk Test.

**Table 3:** Summary of results of additional physical function outcomes.

Outcomes (*N* = 20)	Score Classification at Assessment *N* (%)
Ped-mTNS (*n* = 19)	
Sensory	Normal 10 (53%) Affected 9 (47%)
Functional	Normal 6 (32%) Affected 13 (68%)
Autonomic	Normal 10 (53%) Affected 9 (47%)
Light touch	Normal 6 (32%) Affected 13 (68%)
Pin sensibility	Normal 11 (58%) Affected 8 (42%)
Vibration (*n* = 18)	Normal 16 (89%) Affected 2 (11%)
Strength	Normal 8 (42%) Affected 11 (58%)
Deep tendon reflexes	Normal 5 (%) Affected 14 (74%)
Total score	Normal 5 (26%) Affected 14 (74%)
FP1-6	
Right	Normal 13 (65%) Moderate 6 (30%) Severe 1 (5%)
Left	Normal 12 (60%) Moderate 5 (25%) Severe 3 (15%)
BOT-2 (short form)	Normal 9 (45%) Moderate 7 (35%) Severe 4 (20%)

Score classification ranges for each test are available in Document S1. Abbreviations: BOT-2, Bruininks–Oseretsky Test of Motor Proficiency (2nd edition); FP1-6, Foot Posture Index; ped-mTNS, pediatric-modified Total Neuropathy Scale.

**Table 4:** Descriptive statistics of the BOT-2, FP1-6, and ped-mTNS tests.

Outcome	Overall *N* = 20 Mean (SD) Range
BOT-2 (SF) standard score	42.6 (11.31) 24–65
FP1-6	
Right foot	3.9 (2.85) 0–10
Left foot	5.1 (2.56) 1–10
ped-mTNS (*n* = 19)	
Sensory	0.73 (0.96) 0–3
Functional	0.89 (0.71) 0–2
Autonomic	0.78 (0.89) 0–2
Light touch	1.68 (1.41) 0–4
Pin sensibility	0.57 (0.93) 0–4
Vibration (*n* = 18)	0.16 (0.5) 0–2
Strength	0.63 (0.58) 0–2
Deep tendon reflexes	1.47 (1.46) 0–4
Total score	6.9 (3.45) 0–16

Abbreviations: BOT-2, Bruininks–Oseretsky Test of Motor Proficiency (2nd edition); FP1-6, Foot Posture Index; ped-mTNS, pediatric-modified Total Neuropathy Scale; SF, short form.

**Table 5:** Descriptive statistics for PedsQL scores by subscale.

PedsQL	Baseline (*n* = 19) Mean (SD)	1-Month (*n* = 17) Mean (SD)	2-Month (*n* = 15) Mean (SD)	3-Month (*n* = 20) Mean (SD)
Pain and hurt	65.1 (17)	66.9 (17.6)	65.7 (20.3)	66.9 (16.9)
Nausea	68.2 (18.9)	66.5 (17.1)	65.7 (20.3)	70 (16.1)
Procedural anxiety	58.3 (24.1)	55.9 (23.7)	68.3 (20.7)	66.7 (15.8)
Treatment anxiety	71.1 (22.3)	76 (16.1)	76.7 (22.1)	77.9 (16.9)
Worry	75.9 (23.4)	73 (21.4)	77.8 (20.8)	76.3 (21.3)
Cognitive problems	69.1 (16)	70.5 (14)	72.8 (15.2)	70.8 (18.3)
Perceived physical appearance	81.6 (17.9)	72.1 (22.2)	81.7 (20.2)	76.2 (22.3)
Communication	76.3 (18.3)	65.2 (23.2)	66.7 (24.4)	71.7 (17.2)
Total PedsQL score	70.6 (13.1)	68.1 (11.8)	72 (13.3)	72 (11.4)

Abbreviations: PedsQL, Pediatric Quality of Life Inventory version 4.0.

**Table 6:** ANOVA comparison of PedsQL subscales in relation to the subscale with the lowest mean.

PedsQL Subscales	Mean (SD)	Difference in Relation to Reference
Procedural anxiety	62.2 (21.4)	Lowest mean (reference score)
Pain and hurt	66.2 (17.7)	*p* = 0.279
Nausea	67.7 (17.7)	*p* = 0.186
Communication	70.3 (20.7)	*p* = 0.036
Cognitive problems	70.7 (15.8)	*p* = 0.026
Treatment anxiety	75.4 (19.2)	*p* < 0.001 *
Worry	75.7 (21.4)	*p* < 0.001 *
Perceived physical appearance	77.8 (20.7)	*p* < 0.001 *

* Statistical significance. Abbreviations: PedsQL, Pediatric Quality of Life Inventory version 4.0.

## Data Availability

The original contributions presented in this study are included in the article/Supplementary Material. Further inquiries can be directed to the corresponding author.

## Supplementary Materials

The following supporting information can be downloaded at: https://www.mdpi.com/article/10.3390/pediatric18020036/s1, Document S1: Outcome Measurement Protocol [10,15,20,34,35,36,37,38,39,40,41,42,43,44,46,79,80,81,82,83,84,85,86,87,88,89,90,91,92,93,94,95,96,97,98,99,100]; Figure S1: Distribution by score values, PedsQL; Table S1: Distribution of PedsQL scores for all subscales over time.

## Abbreviations

The following abbreviations are used in this manuscript:

ALL	Acute Lymphoblastic Leukemia
AROM	Active Range of Motion
BOT-2	Bruininks–Oseretsky Test of Motor Proficiency (2nd edition)
CIPN	Chemotherapy-induced Peripheral Neuropathy
FP1-6	Foot Posture Index
HRQOL	Health-related Quality of Life
ped-mTNS	Pediatric-modified Total Neuropathy Scale
PedsQL	Pediatric Quality of Life Inventory version 4.0
PT	Physical Therapy
PROM	Passive Range of Motion
ROM	Range of Motion
6MWT	Six-minute Walk Test
