# Supplementary material for: Physical Therapy Surveillance in Children with Acute Lymphoblastic Leukemia: A Quality Improvement Initiative
Source: Pediatr Rep. 2026 Mar 3;18(2):36. doi: 10.3390/pediatric18020036 (PMC13010702; doi:10.3390/pediatric18020036)
Supplement: Supplementary file 1 [file pediatrrep-18-00036-s001.zip › pediatrrep-4151718-supplementary.pdf]

Document S1. Outcome Measurement Protocol

1. STANDARD PHYSICAL FUNCTION TESTS:

1.1. Ankle Range of Motion (ROM): Passive and active bilateral ankle range of motion is measured using a plastic goniometer for dorsiflexion and plantarflexion. A therapist measures ankle ROM with the child in a sitting position with knees extended.[79] Study participants start with a preconditioning procedure in which they are asked to actively dorsiflex and plantarflex four times, and on the fifth repetition the measurement is taken.[79,80] Normal goniometric measurement of ankle is considered >= 15, and restricted < 15.[10] Clinically, a value of >= 10 is considered functional.[34] Goniometry is a valid and reliable measure for ankle ROM, with an excellent intrarater reliability of ICC= 0.95.[79]

Clinical reference criteria: ROM degrees (Normal >= 15° ; Moderate (functional) >= 10°; Severe < 10°) [34]

1.2. Activity Level: It is measured using the Lansky Play-Performance Scale. This is a tool validated in pediatric cancer patients aged 16 years or younger and is useful to track the disease progression.[35] Parents/ caregivers are asked to rate the child's ability to carry out daily life activities on a scale from 0 to 100. This scale has a good correlation coefficient of 0.71.[35]

Clinical reference criteria: Lanksy score (Normal 80-100; Moderate 50-70; Severe <50) [81]

1.3. Balance: It is measured using the Single Leg Stance test . The child is asked to stand on one leg with open eyes and hands on hips as long as possible. The assessor records the total seconds the participant lasts in the test. In children aged >= 5 years, >10 seconds is considered normal.[36] For children aged <5 years, age norms included in the Peabody Developmental Motor Scale (PDMS-2) are used to grade performance as normal or abnormal. The reliability of this test has been tested in children with cerebral palsy and results showed a moderate to good reliability (ICC 0.56 to 0.99).[82]

Clinical reference criteria: Seconds standing on one leg (In children >= 5 years old Normal >= 10 secs; Affected < 10 secs) [10,36,83]

1.4. Functional Capacity: It is measured using the Six-minute Walk Test (6MWT). According to the American Thoracic Society, the 6MWT is suitable to measure a sub-maximal level of functional exercise capacity,[37] and has been used to assess performance [84-86] and functional capacity,[87,88] in children undergoing cancer treatment. Children are instructed to walk as much distance as possible, without running, and with the possibility of taking rest breaks, for a period of six minutes. The track distance between turning points is 30 meters. Children are instructed and encouraged during the test with standardized phrases similar to those in the Guidelines of the American Thoracic Society.[37] Heart rate and oxygen saturation are taken before and after the test using a pulse oximeter, and the rate of perceived exertion is collected at cessation of the test using the Face Scale or the Borg's 1-10 scale.[89] The total number of laps is collected to calculate the total distance walked. The 6MWT has a high test-retest reliability with an ICC= 0.99 in obese children [90] and 0.98 in children and adolescents with brain injury.[91]

Clinical reference criteria: Predicted distance based on child's characteristics (Normal 80-100%; Moderate 60-79%; Severe <60) – Hospital cut-off values.

1.5. Pain: It is measured using the Wong-Baker Faces Pain Rating Scale. This is a self-report scale that measures pain in children aged 3 to 18 years.[38] The scale comprises a series of 6 faces ranging from happy face (at 0 or 'does not hurt at all'), to a crying face (at 10 or 'the worst pain imaginable'). The child chooses the face and written description that best describes the pain intensity.[92] This scale is widely used in pediatrics and has an excellent validity CI (0.86-0.93).[93]

Clinical reference criteria: **Pain score (Normal** 0 – 2; **Moderate** 4-6; **Severe** 8-10) – Hospital cut-off values.

1.6. Gait: It is assessed using the observational gait analysis. The observational gait analysis is a convenient and suitable alternative when clinical settings lack access to equipment for instrumented gait analysis. The assessment consists of observing the child's walking patterns and identifying possible areas of concern. Four categories are assessed including base of support, foot progression, gait speed, and proximal strength.[39]

Clinical reference criteria: Gait characteristics (Normal = No issues identified; Moderate = Mild deficits; Severe = Moderate and severe deficits (*i.e.,* drop foot)) – Hospital cut-off guidelines.

1.7. Keeling to standing: It is assessed using the Half Kneel to Stand from the Floor test. The assessor observes if the child requires any physical support to stand from the floor.[40]

Clinical reference criteria: Ability to stand from the floor (Normal = Independent; Moderate = Needs upper extremities; Severe = Unable) – Hospital cut-off guidelines.

2. ADDITIONAL PHYSICAL FUNCTION TESTS:

2.1. Motor and Sensory Function: The pediatric modified-Total Neuropathy Scale (ped-mTNS) is used to measure CIPN. This is a validated and reliable pediatric measure that captures information on impairment of the peripheral nervous system through questions on sensory, motor, and autonomic functions.[41] It examines light touch, pin and vibration sensation, distal muscle strength, and deep tendon reflexes.

Light touch sensation: It is assessed using monofilaments. The 2.83 log force monofilament is placed three times on the pad on the index finger, with the participant's eye closed. The participant is asked to indicate each time they feel the stimulus. If the participant is unable to feel the stimulus, monofilaments of bigger size are used until the participant is able to perceive the three stimuli. The lowest level of stimuli perceived is recorded as the light touch sensory threshold. Then, the procedure is repeated on the other hand. The 3.65 log force monofilament is used for the great toe pad of both feet using the same procedure.[41]

Pin sensation: It is assessed using a Medipin™. The assessor demonstrates the test on the participant's forearm and then, with the child's eyes closed, perform the stimulus on the palmar pads of fingers and plantar pads of toes. Four stimuli are given on each area, alternating the "pointy" and "not pointy" ends, and the participant is asked to differentiate the two stimuli given. Scores are considered abnormal if the participant incorrectly identifies at least one stimulus.[41]

Vibration sensation: It is assessed using a handheld Biothesiometer. The voltage will start at 0 with a gradual increase until the participant reports the stimulus ("vibration"). The assessor demonstrates the test on the wrist before performing the stimulus at the distal plantar surface of the great toe and distal palmar pad of the index finger. If the readings are >8 V for the fingers and/or >10 V for the toes, additional tests are performed at the medial malleolus/radial wrist and medial knee/medial elbow until a normal reading is obtained.[41]

Muscle strength: It is assessed on great toe extensors, ankle dorsiflexors, finger abductors, and wrist extensors using the manual muscle testing. Deficits in muscle strength are reported if the muscle group tested is scored <5.[41]

Deep tendon reflexes: Achilles and patellar tendon reflexes are assessed with the Jendrassik Maneuver. Participants are in a seated position with their lower extremities free swinging. Scores are considered abnormal if reflexes are not graded 2+.[41]

Ped-mTNS Scores range from 0 to 4, 0 being no sign or symptom of peripheral nervous system dysfunction. The total score is 32, with higher scores indicating a worser condition. A score of 5 is considered abnormal. This measure has been validated in children between 5 to 18 years of age,[15,41,94] with good internal consistency (α = 0.76), test-retest reliability (ICC = 0.99), and interrater reliability (ICC = 0.98).[41]

Clinical reference criteria: Ped-mTNS Score (Normal <= 4; Affected >4) [41]

2.2. Gross Motor Function: It is assessed using the Bruininks-Oseretsky Test of Motor Proficiency (2nd edition) BOT-2 (Short Form), for children aged 4 to 21.[43,95] This scale is used in children with mild to moderate functional deficits and has been used in pediatric cancer patients.[20,41,96-97] The short form of the BOT-2 comprises eight subtests including fine motor precision, fine motor integration, manual dexterity, bilateral coordination, balance, running speed and agility, upper-limb coordination, and strength.[95] This test will take approximately 30 minutes to complete, depending on the health condition and ability of the child. The interrater reliability, test–retest reliability, a standard error of measurement, and

intercorrelations were established in 1520 subjects for each subtest and age group.[95] The test has an excellent test/re-test reliability, with ICCs ranging from 0.96 – 0.99.[98]

Clinical reference criteria: BOT-2 Score (Normal <=1 SD below mean; Moderate = 1-1.5 SD below mean; Severe >1.5 SD below mean) – Hospital cut-off values.

2.3. Foot posture: It is assessed using the Foot Posture Index (FP1-6).[42] This is a clinical tool to assess the degree to which the child's foot is pronated, supinated, or neutral. The child is instructed to stand still for approximately two minutes while the assessor observes the child's foot and scores 6 categories such as palpation, curvature, position, prominence, congruence, and adduction/abduction. This scale has a validity of (PSI=0.88) with an inter-rater agreement of (Kw = 0.88).[99]

Clinical reference criteria: FP1-6 Score (Normal 0 to +5; Moderate (pronated foot) +6 to +8; Severe (overpronated foot) +9 to +12). Negative values = supinated foot.[100]

3. ADDITIONAL RESEARCH STUDY-SPECIFIC TESTS

3.1. Health-related Quality of Life (HRQL): It is measured using the Pediatric Quality of Life Inventory version 4.0 (PedsQL) – Parent-Proxy, Cancer Module.[44] This tool incorporates eight multidimensional scales, including: Pain and Hurt, Nausea, Procedural Anxiety, Treatment Anxiety, Worry, Cognitive Problems, Perceived Physical Appearance, and Communication. The cancer module comprises 25-27 items, depending on the age group.[44] Higher scores indicate a better HRQL. Participants' caregivers were given the parent proxy to be consistent with the reporting given the variability in ages of children participating in the study. The tool is validated in children aged 2 to 18 years, and the questionnaires are separated in four age groups. The Internal consistency reliability for the PedsQL Cancer Module Scales – parent proxy is average alpha = 0.87.[44]

Clinical reference criteria: PedsQL Score (Normal 81-100; Moderate 61-80; Severe <= 60) [46]

3.2. Self-reported symptoms: It is assessed with a checklist of eight common symptoms and deficits seen in children with ALL at the clinic, including *1)* troubles going up/down the stairs; *2)* challenges with balance (*e.g.,* harder to get up off the floor); *3)* muscle weakness; *4)* pain; *5)* numbness, tingling, or other sensory problems; *6)* Limitations in daily activities (*e.g.,* buttoning clothes, holding a pencil, playing); *7)* changes to how they walk (*e.g.,* heavy feet); and *8)* Not able to keep up with friends/siblings (*e.g.,* slower runner). Parents can also list other symptoms not included in the checklist.

3.3. Feasibility: It is determined based on recruitment rates and completion rates.

1) *Recruitment rates:* Information on recruitment rates are collected to determine the feasibility of the study, including the number of children screened for eligibility, number of children eligible, number of children agreeing to participate, and reasons for refusal to participate in the proposed observational study. This is calculated by dividing the number of children enrolled in the study, by the number of children approached to participate in the study. Screening to recruitment rate will inform on estimated recruitment timelines, required personnel, and budget estimations for planning of a future interventional study.

2) *Completion rates:* Information on completion rates of the study as well as the chosen outcome measures are collected to determine the feasibility of the study. This is calculated by dividing the number of fully completed assessments, by the number of assessments established. Feasibility will be considered as the study completion rate of 80% or more. Also, reasons for no completing assessments and difficulties occurred are documented. This information will inform us about *1)* which outcome measures are feasible for completion at the hospital, and *2)* what considerations should be taken into account when conducting the measurements in a future interventional study.

3.4. Service provision: It is calculated as the number (percentage) of children referred to physiotherapy services compared to the total number seen in clinic. The number of children accessing services, the number of sessions attended, and any reasons for not accessing or not attending sessions are recorded.

Table S1. Distribution of PedsQL scores for all subscales over time

Time point	n	Total score Rank sum	Pain and hurt Rank sum	Nausea Rank sum	Procedural anxiety Rank sum	Treatment anxiety Rank sum	Worry Rank sum	Cognitive problems Rank sum	Physical appearance Rank sum	Communication Rank sum
0	19	683.50	650.50	697.50	627.50	599.50	697.00	633.00	724.50	794.00
1	17	531.50	610.50	578.00	544.00	606.00	569.00	616.50	521.00	539.00
2	15	565.00	575.50	507.00	608.50	561.50	565.00	629.50	600.00	498.50
3	20	776.00	719.50	773.50	776.00	789.00	725.00	677.00	710.50	724.50
***P*-value**		***P*=0.715**	***P*=0.950**	***P*=0.874**	***P*=0.536**	***P*=0.663**	***P*=0.924**	***P*=0.610**	***P*=0.566**	***P*=0.462**

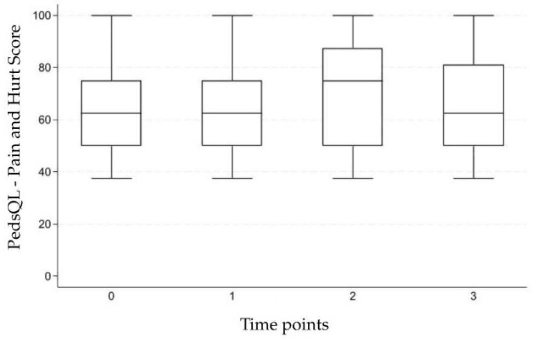
PedsQL - Pain and Hurt Score
0
20
40
60
80
100
0
1
2
3
Time points

(a)

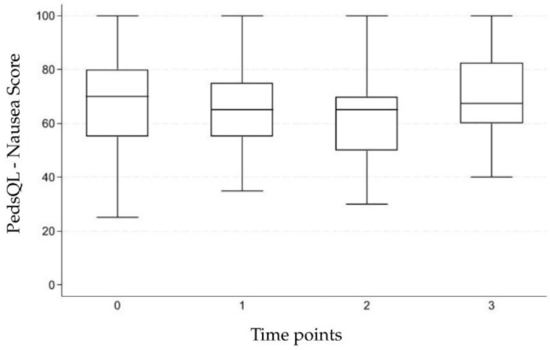
PedsQL - Nausea Score
0
20
40
60
80
100
0
1
2
3
Time points

(b)

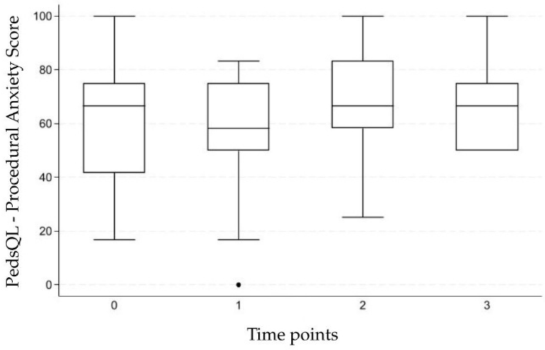
PedsQL - Procedural Anxiety Score
0
20
40
60
80
100
0
1
2
3
Time points

(c)

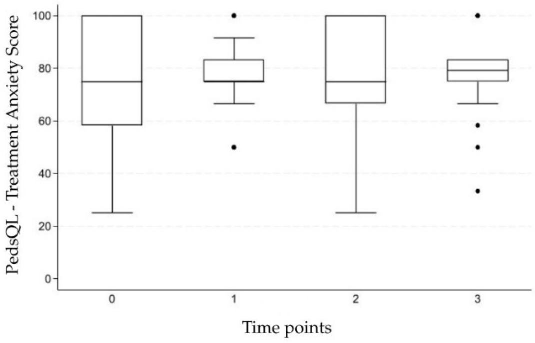
PedsQL - Treatment Anxiety Score
0
20
40
60
80
100
0
1
2
3
Time points

(d)

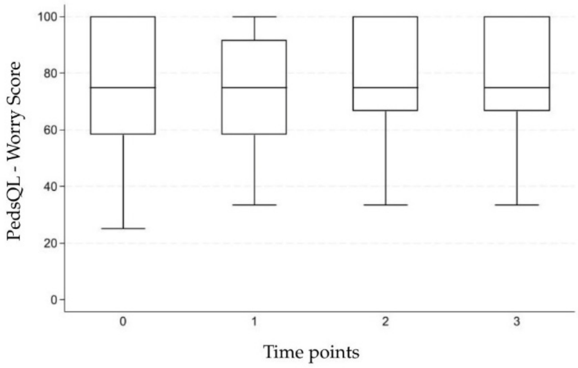
PedsQL - Worry Score
0
20
40
60
80
100
0
1
2
3
Time points

(e)

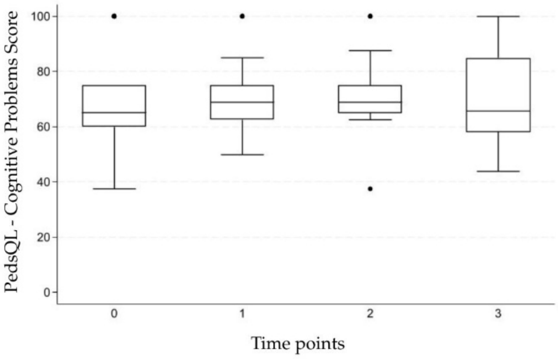
PedsQL - Cognitive Problems Score
0
20
40
60
80
100
0
1
2
3
Time points

(f)

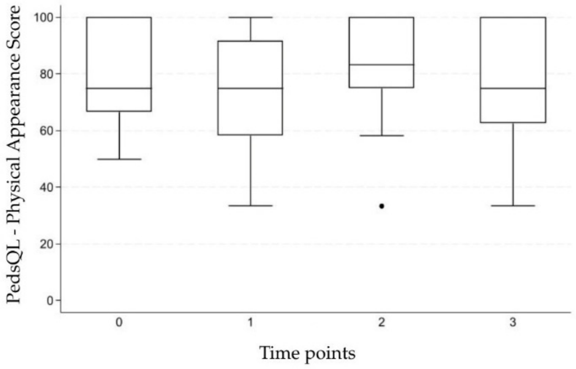

(g)

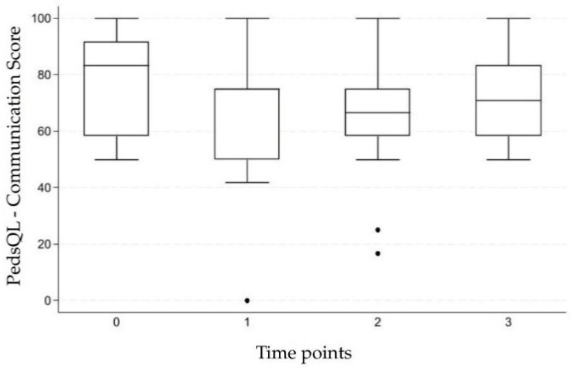

(h)

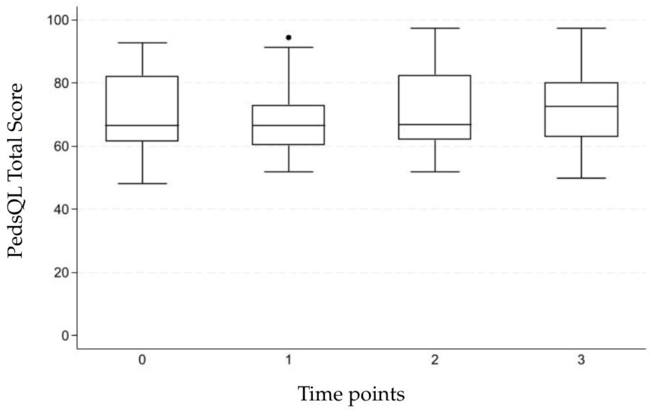

(i)

Figure S1. Distribution of score values by PedsQL subscales: (a) PedsQL Pain and Hurt scores; (b) PedsQL Nausea scores; (c) PedsQL Procedural Anxiety scores; (d) PedsQL Treatment Anxiety scores; (e) PedsQL Worry scores; (f) PedsQL Cognitive Problems scores; (g) PedsQL Physical Appearance scores; (h) PedsQL Communication scores; (i) PedsQL Total scores.
